# Interaction at the primary–secondary care interface: Patients’ and physicians’ perceptions of teleconsultations

**DOI:** 10.1177/20552076221133698

**Published:** 2022-11-28

**Authors:** Ana Rita J Maria, Helena Serra, Maria G Castro, Bruno Heleno

**Affiliations:** 1 Regional Health Administration of Lisbon and Tagus Valley, Comprehensive Health Research Centre (CHRC), Nova Medical School, Faculdade de Ciências Médicas, Universidade NOVA de Lisboa, Lisbon, Portugal; 2 Interdisciplinary Centre of Social Sciences (CICS. NOVA), NOVA School of Social Sciences and Humanities | Faculdade de Ciências Sociais e Humanas, Universidade NOVA de Lisboa, Lisbon, Portugal; 3 58411 Centro Hospitalar e Universitário de Coimbra (CHUC), Coimbra, Portugal; 4 Comprehensive Health Research Centre (CHRC), Nova Medical School, Faculdade de Ciências Médicas, Universidade NOVA de Lisboa; General Practitioner, Regional Health Administration of Lisbon and Tagus Valley, Lisbon, Portugal

## Abstract

**Introduction:**

Previous qualitative research on teleconsultations has focused on synchronous communication between a patient and a clinician. This study aims to explore physicians' and patients' perceptions of the interaction on the interface between primary care and the Cardiology service of a referral hospital through teleconsultations.

**Methods:**

This qualitative study was embedded in an organizational case study concerning the introduction and rollout of a new service model that took place at the point of care. The patients and physicians were recruited for semi-structured interviews until thematic saturation was achieved, between September 2019 - January 2020. The interviews were audiorecorded and anonymized. The transcribed interviews were stored, coded, and analyzed in MAXQDA, following the steps for conventional content analysis.

**Results:**

A total of 29 participants were interviewed. Patients and physicians presented clear views about the role of the GP and the cardiologist and their function in overall structure of healthcare. GPs felt their role was to bring expertise in the patient which could supplement the cardiologists' expertise on the condition. However, GPs had to renegotiate roles in the teleconsultations when they saw themselves in a new situation, together with another physician and the patient.

**Conclusions:**

Our findings suggest that joint teleconsultations can promote continuity of care for patients in the primary/secondary care interface. Active coordination between physicians with delineation of roles throughout primary-secondary care interface is needed to manage selected patients who may benefit the most from shared care.

## Introduction

The primary-secondary care interface is key for the organization of many health care systems. The referral system is the “organizational structure for referring medical problems from generalists to specialists.”^1^ In Portugal, as in Denmark, the Netherlands and the United Kingdom, patients seeking an appointment with a specialist must first be seen by a primary care physician or GP, who acts as a gatekeeper. Gatekeeping has been associated with several benefits such as lower costs, better health outcomes and equity in health care systems.^2,3^ It has been evident there is high variation in the rates of referrals of different GPs^4^ with much debate and analysis.^5–7^ Understanding what affects the referral decision is important for patients to receive the same standard of care, irrespective of location, social status among other factors. The interoperability between different electronic health records platforms at the primary-secondary care interface developed to address the needs of different health care organizations is challenging. Often, physician communication between levels of care requires phone calls, e-mails, letters or using patients as messengers.

Teleconsultations may foster collaborative planning of care, improving continuity and access to care.^8^ As the healthcare needs change from the models in the past to an integrated, longitudinal model for the future, stakeholders from all levels should share common understanding and priorities for integrated care. Telehealth may act as a driver for integration between levels of care and provide lessons for wide-scale future implementation. In the context of evaluating a complex health intervention^9^ specifically the teleconsultations, the primary focus is to explore patients’ and physicians’ perceptions, as it is precisely their choices that determine whether and how a given intervention works.

Previous research on teleconsultations in healthcare support that in selected patients, teleconsultations are noninferior to face-to-face ones in terms of user satisfaction,^10,11^ feasibility,^12,13^ resource use,^14,15^ and clinical outcomes.^16–22^ A systematic scoping review found that primary care patients and clinicians report both positive and negative experiences when using videoconsultations.^23^ We found no previous studies analyzing both patients’ and physicians’ perceptions regarding the implementation process of joint teleconsultations between primary and secondary care within the same context.

Using health information technology (IT) to link GPs and hospital specialists, improving dialog, may optimize the efficient use of specialty resources, and enhance primary care capacity.^24^ It reinforces the patient-centered medicine by supporting GPs in providing longitudinal care for a broader range of conditions and reducing fragmentation of care. It also has the potential to enhance teamwork between GPs and hospital specialists.^25^

Our study aims to explore physicians’ and patients’ perceptions of the interaction on the interface between primary care and the Cardiology service of a referral hospital through teleconsultations.

## Methods

This qualitative study was embedded in an organizational case study of the introduction and rollout of a new service model of teleconsultations that took place at the point of care, taking account the evolving national context. This single case study design^26^ was used to obtain an in-depth understanding of the participants’ perception of a teleconsultation intervention. This type of study design facilitates the understanding of the phenomenon of teleconsultations that is at the same time context-dependent and influenced by the individual's experience and characteristics.^26^ The design was descriptive, and the method was based on semi-structured interviews.

We chose qualitative methods because they help to identify unexpected negative experiences and provide in-depth understanding of how participants’ interactions with interventions produce change.^27^

### Settings

The context for this work is the Portuguese NHS, where general practitioners (GPs) act as “gatekeepers” to secondary care and the communication between levels of care relies on electronic health records (EHRs), store and-forward requests from the GP to the secondary care provider (rarely leading to a dialogue) or on the exchange of letters between the healthcare providers. This study occurred at a time when the Portuguese NHS was seeking to make teleconsultations available whenever appropriate. At the national primary–secondary interface, other initiatives had introduced teleconsultations, albeit scarce information is known from this experience from the available literature.^28^ This project differs from previous experiences, as it allows joint teleconsultations to occur as part of the nationwide EHR platform at the primary-secondary care interface. In this platform, it is possible for cardiologists and GP to read each other's records.

This study took place in six primary care outpatient clinics and the Cardiology service of the referral hospital (Hospital and University Center of Coimbra-General Hospital). The six family health units participating in the project belong to urban or rural practice settings and attend to a total of 59,836 patients. The referral hospital is in Coimbra, at the Central Regional Health Administration, where approximately 1.6 million people live. GPs routinely refer patients to this hospital, as part of their gatekeeping task.

During this study, the GPs could offer teleconsultations to patients when they judged appropriate to refer them to the Cardiology outpatient clinic. Patients and GP participated in teleconsultation at the primary care practice, while the cardiologist participated from the referral hospital. The technology included a videoconferencing tool with synchronous communication, i.e. real time video and audio, capable of producing adequate quality for users to see and to hear each other and for sharing documents (e.g. laboratory results, images). Software was embedded in the National EHR system, a web-based system developed by the Shared Services of the Ministry of Health. Patient, caregiver, and GPs connected from the primary care practice, while cardiologists were at the referral hospital. Communication between the patient, GPs and cardiologists is interactive, allowing the request for additional information for clarification as needed. Participants typically used a computer with a webcam, speaker, wired internet connection and the web browser that is used for the EHRs.

### Participants

Eleven physicians, 14 patients and four caregivers were interviewed. We included patients over 18 years, able to give informed consent, able to speak Portuguese or English, and who had been referred from primary care to cardiology for teleconsultation during the previous two years. Patients could have any medical condition, not requiring acute care, that led to the referral from primary to secondary care. We expected that study participants include mainly chronically ill patients because of their need for frequent care and continuity of care. Patients were recruited for the in-depth interviews from all family health units in person or by telephone, in collaboration with the GPs. When patient participants were accompanied by caregivers, they were also interviewed. We included physicians that participated in at least one teleconsultation for real-time communication when requesting or providing patient-specific information. We excluded patients and physicians who were unable or unwilling to give informed consent for participation or who withdrew at any point of the data collection.

Sample sizes were determined based on ongoing data collection and analysis. Participants were recruited for interviews until thematic saturation was achieved, i.e. when ongoing data analysis yielded no new information or redundancy of theme categories across all sites.

Informed consent was obtained prior to the participation in any study activity. Information was provided by the lead investigator.

### Data collection and data analysis

A semi-structured interview guide for the individual face-to-face interviews was developed based on a review of the literature and discussions among the authors (supplementary material).

Data was collected until there was evidence for data saturation by repetition of themes and the analysis was performed by people with different perspectives, including an expert on qualitative research (sociologist with a special interest in health organizations) and two GPs.

Participants were interviewed in the healthcare facility. The lead investigator (ARJM) conducted the interviews, which were audio-recorded and anonymized. The digital audio recordings were transcribed verbatim by an external transcribing service and the transcripts were validated against the recorded material by the interviewer. All transcribed interviews were analyzed independently by at least two of the researchers to improve reliability. The transcripts were stored, coded and analyzed in MAXQDA (software for qualitative data analysis, 1995–2016, VERBI Software-Consult Sozialforschung GmbH, Version 12.2.1, Berlin, Germany). Data analysis followed the steps for conventional content analysis.^29^ The interviewer and one other researcher coded all data, any discrepancies were discussed until consensus was reached. The coding system was refined until no further codes emerged. After discussion with other team members, codes were categorized. We conducted an open, inductive analysis, starting with open coding. As the objectives of the study are primarily contextual, concerned with understanding people's experiences, attitudes and the nature of the system, the inductive approach keeps a close focus on people's experiences and ideas.

Other ways of thinking critically about data analysis were also employed, including comprehensive data treatment and deviant case analysis. This involved a re-interrogation of the data searching for new themes not covered by the initial data analysis.^30^ We looked for deviant data to contradict our emerging analysis, a process known to add validity.^31^ To ensure confidentiality, interviews were numbered sequentially and the participants’ quotes within findings were identified by the respective numerical code.

## Results

The results from the interviews emphasize how the various actors perceive the interaction on the interface between levels of care with the application of teleconsultations differently and how these perceptions may contribute to organizational changes within the delivery of services. By the time the interviews were conducted (September 2019 to January 2020), all the physicians who were participating in this pilot project were included (Table 1). Although the planned intervention was joint teleconsultations, during the pilot, clinicians supplemented it with remote consultancy between clinicians (teleconsultancy) without the patients being present.

**Table 1.** Characteristics of Study Physicians.

Years in practice	N (%)	GP	Cardiologist	Female	Male	Urban	Rural
< 5 Years	2	1	1	2	0	2	0
6–19 Years	5	5	0	4	1	1	4
> 20 Years	4	1	3	2	2	4	0

These results, while focused on perspectives of physicians and patients, also explored the perspectives of carers (Table 2), who may play a key role in the referral and decision-making process.

**Table 2.** Characteristics of Study Patients and Caregivers.

	Patients (No.)	Carers (No.)
Sex		
Female	7	4
Male	7	0
Age, mean (range), y	71	48
Present during teleconsultation	7	2
Education Level		
< High school grad	13	
High school grad or GED	1	2
Some college or above		2
Available technology		
Computer video camera	1	4
Smartphone	2	2
Mobile phone	8	4
Fixed or landline telephones	13	4

GED: general equivalency degree.

We found two main themes divided into subthemes (Table 3).

**Table 3.** Themes and subthemes related to the teleconsultation's implementation.

Roles - patients - family - physicians - organization
Relationships - doctor–patient communication and rapport - interprofessional relationships

### Roles

*Patients’ role.* Although patients were not involved in the design of this intervention, we found that the patients appreciated being involved in the clinical communication during teleconsultations, as some patients seemed to have little knowledge about their condition. Our findings revealed that patients assume a more passive attitude towards their care, relinquishing control to their doctors, since they seldom spoke proactively about what they could do at an individual level. Some clinicians shared the view that patients assume a passive role, albeit with hope of seeing them more actively participating in the care process, including their formal representation in healthcare planning.

Patient (P) 3: “Doctors know what they can do to us, we must accept. If it's good, we accept everything!”

General practitioner (GP) 6: “The role of the patient is, in fact, very weak. I think it may have to do with a cultural issue. Because people often put their decisions in our hands: ‘what would you do if you were in my shoes?’. This happens a lot: ‘I prefer the doctor to decide for me’, ‘you know what is best for me’. I think this has to do with health literacy. In decision-making, the truth is that the patient also has a certain amount of comfort, but the services are so overwhelmed in the assistance activity and in the clinical activity, that communication with the patient doesn’t happen outside the office.”

Cardiologist (C1): “the patient ends up having a slightly more passive role in this process. Sometimes during the teleconsultation, it will be advantageous for the two doctors to talk with each other.”

*Family's role.* The acceptance of technology is a dynamic process, and the family members have a role when implementing technology. Our results document the family's role organizing and preparing doctor visits, providing, or arranging transportation. The workload associated with this role was reduced with the teleconsultation's implementation.

Caregiver (CG) 14: “It is much better than going to the hospital, without a doubt, much better for us. Even his mobility is also very reduced. We get in here a lot faster than walking through those huge corridors …. Getting an appointment would try anyone's patience.”

*Physicians’ role.* Patients valued the seamless connection between healthcare personnel. Many of the patients and caregivers described a long-term and important relationship with the primary care clinician. They recognized the expert knowledge of the hospital consultant at one field, and that being referred to the cardiologist was also relevant for them. Likewise, we found the belief among the clinicians that the hospital consultant had the “expert” knowledge in a particular field, while the GP is recognized to know the individual patient, including the management of the different health problems, care coordination, understanding of the patients’ family and their circumstances.

CG4: “I very much appreciate the connection between the family doctor, who is closest to the patient, isn't it, and really the specialist who is there in the district hospital. The family doctor has the history, knows what happened.”

GP5: “We know the patient's family context, we know the patient from another perspective, focusing not only the disease, but the patient as a whole. Sometimes it's not possible to write all the information and colleagues will treat a problem and, it can happen, not seeing that problem is also at the base of others. In the example of cardiology, they focus the cardiac part, but then we must watch the rest.”

Cardiologist (C) 1: “It is not only the fact that we want to receive the patients as early as possible, but we also want to receive those that really need us. We know that our colleagues GPs also have this awareness, that some of the patients sent to us we don’t need to see. We really want to organize this whole communication system so that we would effectively receive those patients who need it, because it would allow us to give a much quicker response. If we could only have the patients that need us here, we would give [snapping fingers] answer I would say almost immediately. I think that everyone would be satisfied, of course, GPs, but especially the patients. The problem now is that we live here crushed, as well as our colleagues surely crushed, with cases, and some of them shouldn’t be here.”

Patients, GPs, and cardiologists presented clear views about the role of the GP and the cardiologist and their function in overall structure of healthcare. We found that GPs felt empowered in addressing more of their patients’ needs.

GP4: “We take the doubts out of the medication, we advise according to the opinion of the cardiologist I even think it's better, because when they [patients] go to the consultation they often do not understand. It is explained by the GP to the patient what was decided. We explain everything in detail, what will change, what has been decided.”

However, when physicians saw themselves in a new situation together with the patient, they had to renegotiate roles. We found that the patients’ presence during joint teleconsultations appeared to create a degree of discomfort for some GPs, mainly because it caused a lack of clarity for clinicians about roles and conduct in these consultations. These GP favored teleconsultations without the patient being present, where they could have a peer discussion with the cardiologist.

GP1: “There was a conflict of roles, and I think this was the hardest part in the teleconsultation and that made me even uncomfortable. Because as the patient's GP I was placed in a position like a clinical examiner or a transcriber of diagnostic tests, towards another doctor who seemed wise in everything. This makes a relationship difficult.”

Other GPs felt they could have their role as patient advocate, clarifying aspects of the cardiology teleconsultation that the patient may have not understood. Cardiologists expressed positive attitudes towards a shared methodology with the GP for the patient care.

C4: “Often we want to start anticoagulants, following the guidelines, but then, as a whole, maybe it's not such a good idea. Because we need to be vigilant, maybe the patient doesn’t know how to say this or that, and the GP, in the end, knows everything.”

*Organization's role.* Some patients and caregivers interviewed suggested governmental resource commitment and infrastructural and healthcare human capital development for the future.

P6: “If the services are well equipped with everything that is needed, I have the impression that the [communication] interferences should not take place!”

C4: “A team in this case would include the doctor, nurse, psychologist, assistant. Like a multidisciplinary team that would be monitoring and even watching the needs.”

P2: “If the good healthcare technicians do not find the appropriate conditions here, they will look for them elsewhere. That is why we are lacking healthcare professionals in our country. Here they are not paid as much as they are abroad.”

### Relationships

*Doctor–patient communication and rapport.* Physicians felt that the doctor–patient relationship will likely retain a central role in how care is perceived, regardless of the mode of delivery (e.g. virtual or face-to-face). On the other hand, patients had mixed feeling. While some, welcomed the remote interaction, others expressed potential obstacles in the doctor-patient communication with this mode of healthcare delivery, with diminished doctor–patient rapport when trouble with the technology happened (e.g. absent video).

GP2: “The patient has a greater confidence in what is transmitted to him, because he knows that a discussion with the cardiologist happened. They end up feeling more assured as well.”

P9: “I don't like it at a distance, it was a strange thing, because I wasn’t seeing the doctor! I like to see the person. The doctors were speaking to each other, and I was listening. Then the cardiologist spoke to me, but I didn’t see her, she just greeted me.”

P6: “[the doctor-patient relationship] I think it's good. I didn’t see the cardiologist, but if I saw I would be talking to the cardiologist as I'm talking to you, it's the same thing.”

Also, our findings suggest that primary care encounters and relationships can have an impact on patients’ experiences with clinicians and clinical services in other settings, as one patient and the respective caregiver mentioned a lack of careful listening and understanding of the patient's distress by their GP. If patients felt they had poor GP-patient relationship, they concluded that the GP could not be a proper advocate in their behalf during a conference between the GP and the cardiologist.

CG13: “this doctor neither checks the blood tension, nor listens to the person. So I don’t know what he can say to the other side, and what can the other side solve. In order to work well, there must be people with the capacity to clarify what the person has and concretize in terms of medications or tests.”

*Interprofessional relationships.* The reach of teleconsultations into primary care offers several advantages including enabling a closer relationship and shared knowledge between clinicians and speedier feedback of information to patients.

GP6: “Teleconsultation makes us visible. We stop being transparent and invisible, we no longer have a written name … and we start to have a face.”

C4: “The GP was really concerned with solving the patient's problem in a timely manner.”

Our findings highlight the need of respecting collaborative networks between professionals in the referral processes.

C4: “This project has everything to benefit mainly the patient, and also for us to do more and better, because I think that what makes sense is that we may grow with each other. The GP feels much more at ease in asking questions.”

GP1: “If we increase the quality of the referrals, we will automatically increase our technical-scientific quality. This will make patients recognize us with more capacity, improving the relationship of trust.”

Our results elucidate the ways in which joint teleconsultations created interactional difficulties in practice. The nature of these difficulties may be related with differences between the style of the diverse clinicians involved, related with many dimensions of each specialty.

GP1: “There was a very big divergence between what we promote through motivational interviewing and what is the relationship established at the hospital, which is a distance relationship, of paternalism.”

Physicians expressed positive attitudes to the development of professional relationship between them, describing the advantages of the interactive learning process.

C2: “This type of intervention brings us closer to the GP. Because when we only receive information via computer and we don't even know the doctor, we don't have the same connection. When we start to do telemedicine, that connection becomes closer, so we get a better understanding of how that doctor interprets, both the symptoms, the signs, and the data of the objective tests. Along the process I think it will be very useful, the confidence that is acquired, and the learning that we both receive and transmit.”

## Discussion

The implementation of teleconsultations at the interface between primary care and the Cardiology outpatient clinic influenced roles and relationships. Patients and physicians presented clear views about the role of the GP and the cardiologist and their function in overall structure of healthcare. GPs felt their role was to bring expertise to the patient which could supplement the cardiologists’ expertise on the condition. However, GPs had to renegotiate roles in the teleconsultations when they saw themselves in a new situation, together with another physician and the patient.

Patients valued how telemedicine promoted a seamless connection between their physicians. Nevertheless, they had mixed opinions about remote interaction with physicians, as they recognized the value of face-to-face consultations in doctor-patient communication and rapport. Physicians expressed positive attitudes towards teleconsultations as an instrument to improve coordination of care, mentioning closer interprofessional relationships, better knowledge exchange and speedier feedback of information to patients. Some physicians favored teleconsultations without the presence of the patient, where they could use it as an interactive learning process through peer discussion.

By the time we ended the data collection, the project was expanding to more primary care practices, just before the beginning of the COVID-19 pandemic. For health systems planning to implement teleconsultations, this pilot project experience can offer important lessons in anticipating challenges with users’ expectations and problems emerging with health IT adoption to generate transferable knowledge to routinizing this service model. At a time when the drive to expand teleconsultations in primary care has been given new impetus and support by the COVID-19 pandemic, this work provides a knowledge base useful to other countries which intend to implement telemedicine into their healthcare systems and is the first study addressing in-depth both patients’ and physicians’ perspectives on teleconsultations between primary and secondary care both at the same case study.

### Roles

Patients might often be unaware of the extent to which they are able to influence events concerning them, such as their impact on the decision to referral. As reported elsewhere, we found that both the relationship with the GP and the experience of having a referral to the cardiologist^32^ is relevant for the patient, translating into a seamless connection between healthcare personnel. Also, some clinicians perceived that patients assume a passive role, consistent with a previous study,^33^ albeit with hope of seeing them more actively participating in the care process, including their formal representation in healthcare planning. Although we found that patients assume a more passive attitude toward their care, we recognize that the competencies relevant to medical decision-making and the relationship between health literacy and self-reported patient involvement through teleconsultations pose an interesting gap of knowledge.

Family's role is important, and technology facilitates an “operative” role (e.g. arrangement of transportation for appointments); this is relevant for patients living in remote locations, but especially for patients who receive support in activities of daily living. Additionally, for older patients, specific health events can trigger the need of using technology,^34^ and especially grandchildren can positively influence the acceptance of technology.^35^

Our findings are in line with others, as clinicians need time to develop and establish new roles in work processes that come with the concept of teleconsultations.^36,37^ However, new tasks that are introduced when using new technologies, such as sharing tasks (e.g. performing additional testing requested by the cardiologists, or explaining the patient the results of the joint teleconsultation in nontechnical language) lead to changes that may threaten and disrupt spatial, professional and organizational orders in the work and organization.^38^

Cardiologists expressed positive attitudes toward a shared methodology with the GP for patient care, in line with another study,^39^ recognizing a crucial role for the GP who already has an established relationship with the patient and is aware of all the relevant psychosocial context. However, we found that the patients’ presence during joint^40^ teleconsultations appeared to create a degree of discomfort for some GPs, mainly because it caused a lack of clarity for clinicians about roles and conduct in these consultations, which is reported elsewhere.^41^ This may be due to the fact that different forms of communication involve a reconfiguration of relations and interactions for clinicians and patients, as well as between all these actors and the organization itself, which in turn leads actors collectively to learn, innovate and adopt new modes of communication and work. We propose that enlarging collaborative strategies between providers through teleconsultations should help improve the integration of patients’ care.

Innovative technological advances in health care are inevitable and our results drawn attention to the contribution of telehealth to the increasingly complex healthcare roles, which is consistent with previous research.^42,43^ Healthcare professionals are managing boundaries and identities that the introduction of telehealth has brought about, requiring them to develop a shared understanding and to re-negotiate and delineate their own roles and responsibilities.

### Relationships

Given the paucity of research on doctor–patient communication and patients’ experiences with real time teleconsultations, it is unknown whether GPs are aware of their patients’ preferences. Patients’ perceptions of quality of care appear to be strongly connected to the quality of the doctor–patient interpersonal engagement.^44–47^ Our previous work described technical issues using teleconsultations, as communication may become less effective, and patients recognized the value of face-to-face consultations in communication and relational issues.^48^

As reported in previous research, clinicians who use videoconferencing together with their patients, should ensure that patients are able to understand what is being discussed^49^ and should involve the patients in clinical communication.^50–52^ Primary care relationships can have an impact on patients’ experiences with clinicians, which is an important factor for the assessment on whether to trust the doctor.^53^

Trust seems to be a vital factor for the patient's comfort in using e-mediated communication technology in the first place, and then, the technology might have the potential to stimulate the building of patient-doctor relationships in alternative ways.^54^ Realizing the potential of the revolution in electronic communications will require the strengthening of clinician ethics for generosity and collaboration, centered on people, as authors reflect.^55^ Our findings suggest such hope may be warranted: the reach of teleconsultations into primary care offers potential advantages including shared knowledge between clinicians and speedier feedback of information to patients.

The establishment of personal relationships for the development of trust is an important element for the ability to work together and for telehealth success.^56^ Our findings highlight the need of respecting collaborative networks between professionals in the referral processes. This means that, not only there is evidence of the “practical” elements of integration such as information flows, but also, there is evidence of the development of a “genuine dialogue” or shared culture described in terms of the way people interacted with one another, consistent with previous research.^57–59^ Because of the collaborative nature of referral communication and the mutual knowledge involved, better relationships between GPs and hospital consultants may contribute to bridge gaps in ways that asynchronous EHRs cannot.

We found that GPs and cardiologists expressed positive attitudes to the development of professional relationship between them, describing the advantages of the interactive learning process. This is consistent with previous research, emphasizing that considering each other's work situation is essential for a balanced communication to happen.^60,61^

However, in keeping with other research about interactional aspects of telemedicine,^41,62^ our analysis elucidates the ways in which joint teleconsultations created interactional difficulties in practice. Besides, the professional duty of providing care prevails in minds of care providers and takes precedence over dealing with administrative coordination of care. This finding may best be understood in terms of a “professional-bureaucratic work conflict.”^63,64^

### Strengths and limitations

The major strength is the inclusion of patients, caregivers, GPs and hospital consultants. To our knowledge, it is the first case study to explore the experiences of all these participants with joint teleconsultations, and the first in-depth description of joint teleconsultations’ implementation in Portugal. The use of an interviewer who was independent of the pilot project management contributed to the overall robustness of the findings. The use of an interview guide provided a uniform record to increase rigor. Interviews were conducted by one researcher, which favors questioning consistency and rapport with participants. Analysis by a multidisciplinary team facilitated the consideration of alternate interpretations of findings. There are limitations to this study. We did not interview non-adopter clinicians and patients, who may have different views on teleconsultations. The data used in this analysis is from the perspective of GPs and cardiologists, but knowledge and attitudes regarding teleconsultations of health professionals from different specialties might differ. Additional studies including a diverse set of specialties are needed to explore their perspectives further on primary-secondary care communication and expand generalizability. Also, this case study took place in a single NHS geographic region, and thus our findings may not reflect co-management practices and attitudes in all NHS primary care practices as well as in private practice. Notwithstanding, teleconsultation's pilot implementation is expanding to more practices and the referral process between primary healthcare centers and secondary care is generally similar across the country. The purpose of this study was to obtain context-dependent knowledge to help program leaders better understand the important role of context in the implementation of teleconsultation.

There are limits to the transferability of our findings. Our focus on patients with cardiovascular disease meant that the included patients were older and most presenting multiple chronic conditions. Therefore, efforts should be made to identify populations and visit purposes most appropriate to integrate telehealth visits between levels of care.

Further work involving both patients and practitioners is needed to fully appreciate the consequences of a shift away from physical presence in consultations, and the implications of online systems for the quality of consultations.

More research is needed to expand the knowledge on process outcomes, such as the quality of relations and how the different actors need to renegotiate their roles in the teleconsultations, to provide a better understanding of the normative aspects of integrated health services delivery.^65^

Our work highlighted perceptions of the interaction through teleconsultations that are important the patients and caregivers and they should further contribute to the development of patient-centered outcome measures. Family members’ attitudes might contribute to better understand the patients’ perceived need to adopt the intervention, as they might be sensitive to the caregiver's influence.

Patients were not involved in the design of this intervention. There are challenges when engaging patients in developing healthcare services, but the active engagement of participants in interviews about their healthcare delivery suggests that patient involvement could potentially help to improve care models and enhance their uptake. We hope that our findings will prompt more telehealth strategies designers and implementers to develop meaningful collaborations with patients.

Educating both GPs and cardiologists in the request and provision of teleconsultations may improve their quality and may promote their broader implementation. Future work should also examine the intersubjective experience of physical examination from both patients’ and physicians’ perspectives, perhaps based on direct observation.

## Conclusions

Our study suggests that when physicians saw themselves in a new situation together with the patient, they had to renegotiate roles. Active coordination between physicians with delineation of roles throughout primary–secondary care interface is needed to manage selected patients who may benefit the most from shared care. Our findings suggest that joint teleconsultations can promote continuity of care for patients in the primary/secondary care interface. However, as patients recognize the value of face-to-face consultations in doctor–patient communication and rapport, a proposed combination of both modalities (remote and face-to-face) may be the most appropriate model of care.
